# Ginsenosides Rb3 and Rc Exhibit Anti-Amoebic Activities Against *Naegleria fowleri*, the Etiological Agent of Primary Amoebic Meningoencephalitis

**DOI:** 10.3390/ph19040573

**Published:** 2026-04-02

**Authors:** Thu Hằng Nguyễn, Hương Giang Lê, Tuấn Cường Võ, Minkyoung Cho, Byoung-Kuk Na

**Affiliations:** 1 Department of Parasitology and Tropical Medicine, Institute of Medical Science, Gyeongsang National University College of Medicine, Jinju 52727, Republic of Korea; thuhang3399@gmail.com (T.H.N.); gianglee291994@gmail.com (H.G.L.); vtcuong241@gmail.com (T.C.V.); mcho@gnu.ac.kr (M.C.); 2 Department of Convergence Medical Science, Gyeongsang National University, Jinju 52727, Republic of Korea

**Keywords:** ginsenoside Rb3, ginsenoside Rc, *Naegleria fowleri*, anti-amoebic activity, therapeutic candidate

## Abstract

**Background/Objectives:** *Naegleria fowleri* is an opportunistic pathogen causing primary amoebic meningoencephalitis (PAM), a fatal neuroinflammatory disease with a high mortality rate of over 97%, in humans. Currently, there are no approved therapeutics for PAM, underscoring the urgent necessity of developing effective and safe drugs. This study aimed to evaluate the potential of ginsenosides Rb3 and Rc as alternative or supplementary drug candidates for PAM by assessing their anti-amoebic activities against *N. fowleri*. **Methods:** Anti-*N. fowleri* activities of ginsenosides Rb3 and Rc and their cytotoxicity to C6 glial cells were evaluated by cell viability assay. The underlying anti-amoebic mode of action of Rb3 and Rc was analyzed by a series of assays for apoptosis–necrosis, TUNEL, intracellular reactive oxygen species (ROS), mitochondrial dysfunction, ATP production, caspase-3, and autophagy. The expression profiles of apoptosis- and autophagy-related genes were also analyzed. **Results:** Rb3 and Rc effectively induced death of *N. fowleri* trophozoites with IC_50_ values of 94.71 ± 1.63 μM and 126.99 ± 1.88 μM, respectively. However, Rb3 and Rc showed no significant cytotoxicities against C6 glial cells, suggesting their selective anti-*N. fowleri* activities. Typical apoptosis signals, such as apopxin staining and DNA fragmentation, were detected in amoebae upon treatment with Rb3 or Rc. These two ginsenosides enhanced ROS production and induced mitochondrial dysfunction in the amoebae. Enhanced caspase-3 activity and autophagy formation were also identified in amoebae treated with Rb3 or Rc. **Conclusions:** These results provide the first evidence that ginsenosides Rb3 and Rc induce apoptosis-like programmed cell death in *N. fowleri*, suggesting that they are potential candidates in developing novel therapeutic strategies against PAM.

## 1. Introduction

*Naegleria fowleri* is a free-living amoeba, but it can infect humans opportunistically and cause primary amoebic meningoencephalitis (PAM), a fatal neuroinflammatory disease [1]. This amoeba has been typically found in various environments, such as soils, freshwater, and artificial freshwater systems, including swimming pools, tap water, and recreational facilities [2]. Infections usually occur when individuals are exposed to freshwater contaminated with *N. fowleri*, allowing the amoeba to enter the human body through the nasal route. Inhaled amoeba penetrates the nasal mucosa, moves along the olfactory nerve, and reaches the olfactory bulbs [3]. It then travels to the brain through the cribriform plate and triggers massive destruction of brain tissues and severe inflammation cascades in infected foci, eventually leading to death [2,4,5,6].

About 500 global PAM cases have been reported, with the highest numbers in the United States of America, Pakistan, and Australia. However, PAM cases are not limited to specific countries or continents [7]. Although PAM is a rare disease, it progresses rapidly, resulting in death less than two weeks after the initial exposure, with a high mortality rate of over 97% [8,9]. Actual global PAM cases might have been largely underestimated due to the diagnostic difficulty and the absence of a systematic surveillance system [8]. Given the increasing incidences of PAM in recent years and climate change-associated environmental shifts expanding the ecological range of *N. fowleri* [8,9,10], there is an increasing concern for the global spread of this deadly amoebic disease.

Early diagnosis and prompt treatment of PAM are essential to reduce lethality. Amphotericin B (AmpB) and miltefosine (MF) have been recommended as the drug of choice to treat PAM; however, their therapeutic efficacy is limited [1,11]. Combination approaches of these two drugs with antibacterial agents have also been explored [1,9]. However, these treatment options have not demonstrated significant therapeutic effects [12,13], emphasizing an urgent need to develop novel therapeutics for PAM. Many approaches to discovering anti-*N. fowleri* compounds in natural resources as alternative PAM drugs have been studied recently. In particular, plant-origin or plant-derived components have received considerable attention [14,15,16,17,18,19,20,21,22,23,24]. Plant-derived natural compounds are considered safe, as they have been used throughout human history to manage or treat various diseases, including infectious diseases [25]. The promising anti-amoebic or amoebicidal effects of several natural compounds or plant extracts against *N. fowleri* have been demonstrated, raising expectations for their potential as novel treatments for PAM. However, further investigations are needed to determine their biological safety and clinical effectiveness.

Ginseng, a root of the Genus *Panax*, has been used in traditional medicine in many countries for centuries due to its promising pharmacological activities [26]. Ginseng contains a diverse array of bioactive and pharmacologically functional components, including fatty acids, ginsenosides, peptides, polysaccharides, polyacetylenes, and polyacetylenic alcohols. These bioactive constituents exhibit considerable preventive and therapeutic effects against various human diseases, including hepatic, cardiovascular, metabolic, inflammatory, and neurological diseases [27,28,29,30,31]. The antimicrobial properties of ginseng and its bioactive compounds have also been elucidated, suggesting their potential as sources for antimicrobial therapeutics [26,32,33]. However, studies on their antimicrobial activities are limited.

In the present study, we explored anti-amoebic activities of 11 bioactive ginsenosides against *N. fowleri* trophozoites, and identified that ginsenosides Rb3 and Rc exhibited promising anti-*N. fowleri* activities by inducing apoptosis-like programmed cell death (PCD) in the amoeba.

## 2. Results

### 2.1. Rb3 and Rc Exhibit Anti-N. fowleri Activities

**Figure 1:**
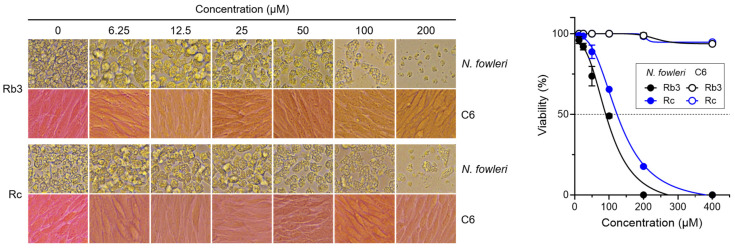
Anti-*N. fowleri* activity assay. Representative microscopic images from three individual experiments show morphological changes in *N. fowleri* trophozoites and C6 glial cells upon treatment with Rb3 or Rc. Viabilities of amoebae and C6 glial cells are presented as a percentage relative to the negative control not treated with Rb3 nor Rc.

A set of 11 ginsenosides was screened for anti-amoebic activities against *N. fowleri*. Among these ginsenosides, Rb3 and Rc showed anti-*N. fowleri* activities with high SI values of 10.17 and 7.87, respectively (Table 1). IC_50_ and IC_90_ values of Rb3 against *N. fowleri* were 94.71 ± 1.63 μM and 170.96 ± 0.71 μM, respectively. IC_50_ and IC_90_ values of Rc against the amoeba were 126.99 ± 1.88 μM and 229.23 ± 0.31 μM, respectively. Neither Rb3 nor Rc exhibited significant cytotoxicity to C6 glial cells, with CC_50_ values of 963.23 ± 32.23 μM and up to 1000 μM, respectively. Although compound K (CK), Rg3, and Rh2 also exhibited anti-amoebic activities against *N. fowleri*, they showed substantial cytotoxicities to C6 glial cells, resulting in SI values lower than 4. The other six ginsenosides, Rg1, Rg2, Rh1, Re, Ro, and F1, did not show any prominent anti-*N. fowleri* activities. Therefore, Rb3 and Rc were selected for further experiments. Rb3 and Rc induced prominent morphological changes, such as size reduction and shape deformation, of *N. fowleri* trophozoites in a dose-dependent manner (Figure 1). A similar morphological alteration was also observed for amoebae treated with MF. Meanwhile, no morphological changes were observed for C6 glial cells upon treatment with either Rb3 or Rc. Viability assay revealed that Rb3 and Rc caused remarkable reductions in the viability of *N. fowleri* trophozoites, but they did not affect the viability of C6 glial cells (Figure 1). These results indicate that Rb3 and Rc possess selective anti-*N. fowleri* activities without notable cytotoxic effects on C6 glial cells.

### 2.2. Rb3 and Rc Induce Apoptotic Cell Death in N. fowleri Trophozoites

**Figure 2:**
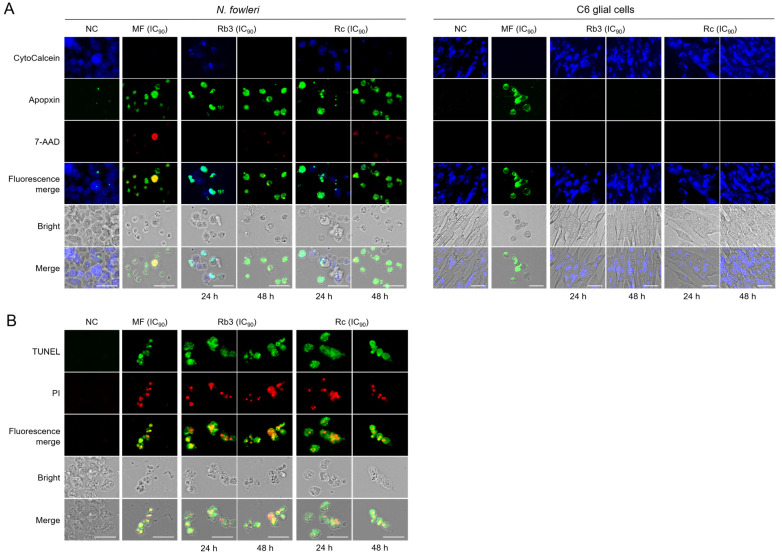
Apoptosis/necrosis and TUNEL assays. (**A**) Apoptosis/necrosis assay. Images are representatives of cell populations in three individual experiments. NC, negative controls. Amoebae or C6 cells treated with MF (IC_90_) were used as positive controls. Size bar: 25 μm. (**B**) TUNEL assay. Green fluorescence indicates DNA fragmentation, and PI (red fluorescence) was used for counterstaining. Images are representatives of amoeba populations from three individual experiments. NC, negative controls. MF was included as a positive control. Size bar: 25 μm.

*N. fowleri* trophozoites treated with Rb3 or Rc showed strong green fluorescence of apopxin, indicative of apoptotic events. Rb3 and Rc increased green signals in the amoebae in a time-dependent manner (Figure 2A). Weak red fluorescence by 7-AAD, a marker for late apoptosis or potential necrosis, was also detected in a few amoebae upon treatment with Rb3 or Rc (Figure 2A). In contrast, Rb3 and Rc did not induce apoptotic or necrotic cellular events in C6 glial cells, indicating no effect against the cells (Figure 2A). To further analyze the association of apoptosis events in *N. fowleri* deaths caused by Rb3 or Rc, a TUNEL assay was performed. Green fluorescence indicating DNA fragmentation, a hallmark of the apoptotic event, was detected in *N. fowleri* trophozoites treated with Rb3 and Rc (Figure 2B). Red fluorescence of PI was also detected in Rb3- or Rc-treated amoebae, implying potential plasma membrane damage. These results suggest that Rb3 and Rc induce specific apoptotic PCD in *N. fowleri* trophozoites.

### 2.3. Rb3 and Rc Enhance Intracellular ROS Production in N. fowleri Trophozoites

**Figure 3:**
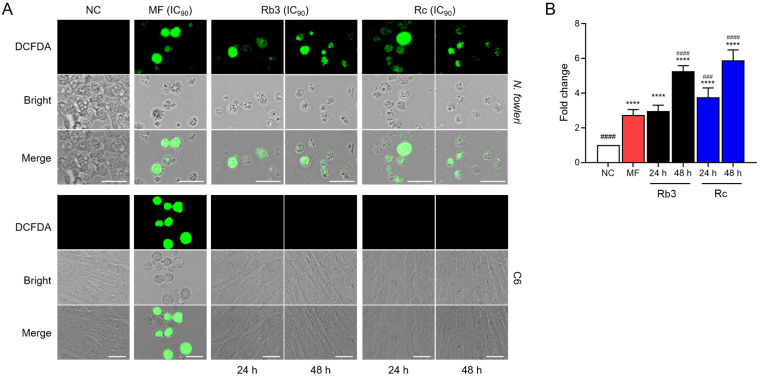
Intracellular ROS assay. (**A**) Fluorescence microscopic analysis. Representative cell images from three individual experiments were presented. Size bar: 25 μm. (**B**) Fluorometric analysis. Fold-change in DCFDA fluorescence intensity was increased in *N. fowleri* trophozoites treated with Rb3 or Rc. NC, negative controls. MF was included as a positive control drug. **** *p* < 0.0001 compared to NC. ^####^ *p* < 0.0001 and ^###^ *p* < 0.001 compared to MF.

Significant time-dependent increases in intracellular ROS were detected in *N. fowleri* trophozoites upon treatment with Rb3 or Rc (Figure 3A,B). Although MF induced intracellular ROS production in both *N. fowleri* trophozoites and C6 glial cells, Rb3 and Rc selectively increased intracellular ROS in *N. fowleri*, without affecting C6 glial cells (Figure 3A).

### 2.4. Rb3 and Rc Induce Mitochondrial Dysfunction in N. fowleri Trophozoites

**Figure 4:**
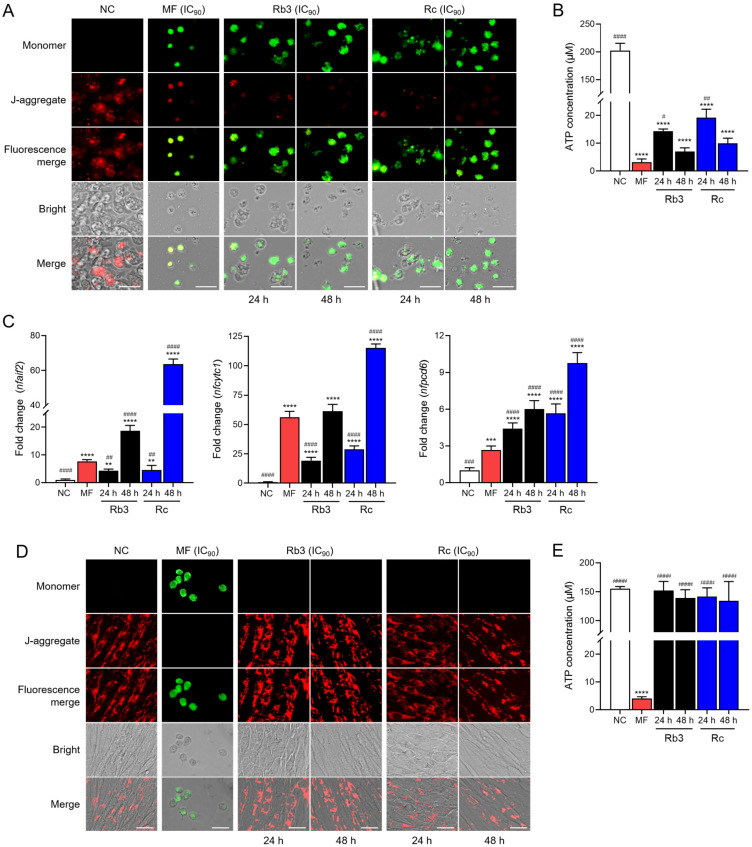
Mitochondrial dysfunction in *N. fowleri* trophozoites. (**A**) Changes in mitochondrial membrane potential. In amoebae treated with Rb3 or Rc, green fluorescence of the JC-1 monomer, indicating mitochondrial membrane potential disruption, was remarkably increased. Images are representatives of cell populations observed in three individual assays. Size bar: 25 μm. (**B**) ATP assay. A significant decrease in ATP production was detected in amoebae treated with Rb3 or Rc. **** *p* < 0.0001 compared to NC. ^####^ *p* < 0.0001, ^##^ *p* < 0.01, and ^#^ *p* < 0.1 compared to MF. (**C**) Expression profiles of apoptosis-related genes. Gene expression levels were analyzed by qRT-PCR. The fold-change in gene expression was normalized against *nfgapdh* as an internal control gene. Amoebae treated with MF (IC_90_) were used as positive controls. **** *p* < 0.0001, *** *p* < 0.001, and ** *p* < 0.01 compared to NC. ^####^ *p* < 0.0001, ^###^ *p* < 0.001, and ^##^ *p* < 0.01 compared to MF. (**D**) Mitochondrial membrane potential assay in C6 glial cells. C6 glial cells treated with Rb3 or Rc showed only red fluorescence, implying JC-1 aggregates, similar to signals observed in C6 glial cells without treatment with ginsenoside (NC). Size bar: 25 μm. (**E**) ATP assay. No significant decrease in ATP production was observed in C6 glial cells treated with Rb3 or Rc. **** *p* < 0.0001 compared to NC. ^####^ *p* < 0.0001.

Rb3 and Rc increased green fluorescence of monomer signals, indicating mitochondrial damage in *N. fowleri* trophozoites in a time-dependent manner, while red J-aggregate fluorescence signals, implying depolymerization of mitochondrial membrane potential, decreased accordingly (Figure 4A). ATP production also significantly declined in amoebae treated with Rb3 or Rc (Figure 4B). qRT-PCR analyses of genes associated with mitochondrial dysfunction, such as *nfaif2*, *nfcytc1*, and *nfpcd6*, suggested significant upregulations of these genes in *N. fowleri* treated by Rb3 or Rc (Figure 4C). Meanwhile, Rb3 and Rc did not induce mitochondrial damage in C6 glial cells (Figure 4D,E). These results suggest that Rb3 and Rc induce mitochondrial damage and dysfunction in *N. fowleri* trophozoites, without affecting mitochondrial functions in C6 glial cells.

### 2.5. Rb3 and Rc Increase Caspase-3 Activities in N. fowleri Trophozoites

**Figure 5:**
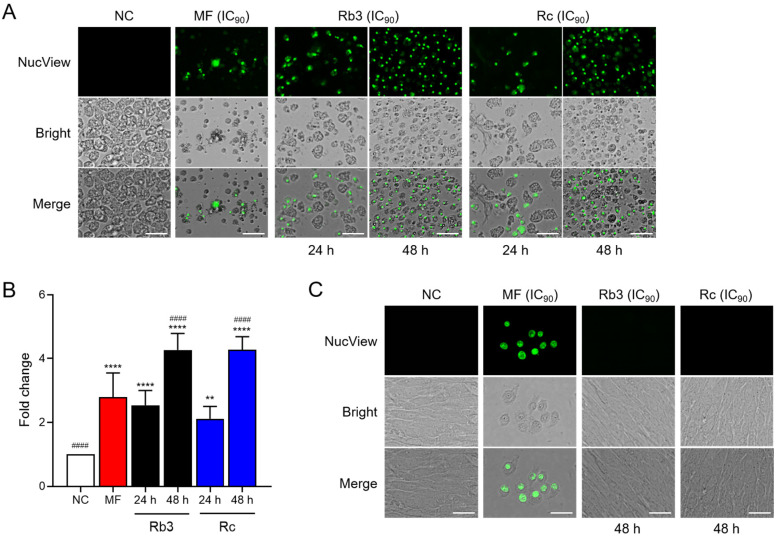
Caspase-3 assay. (**A**) Fluorescence microscopic analysis. Green caspase-3 fluorescence was increased in amoebae treated with Rb3 or Rc in a time-dependent manner. NC, negative control amoebae were not treated with ginsenoside. MF (IC_90_) was included as a positive control. Representative cell images from three independent assays were presented. Size bar: 25 μm. (**B**) Fluorometric analysis. Fold-change in NucView fluorescence is shown. **** *p* < 0.0001, and ** *p* < 0.01 compared to NC. ^####^ *p* < 0.0001 compared to MF. (**C**) Assay for C6 glial cells.

Treatment with Rb3 or Rc led to time-dependent increases in caspase-3 activities in *N. fowleri* trophozoites (Figure 5). A similar increase in caspase-3 activity was also detected in amoebae treated with MF, while no detectable caspase-3 activity was observed in negative controls.

### 2.6. Rb3 and Rc Activate the Formation of Autophagosomes in N. fowleri Trophozoites

**Figure 6:**
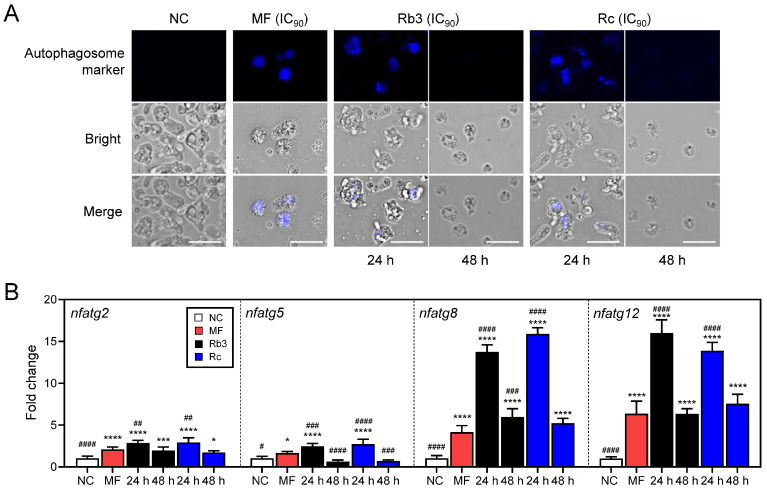
Autophagy assay. (**A**) Fluorescence microscopic analysis. Blue fluorescence indicating autophagosomes was detected in *N. fowleri* trophozoites treated with Rb3 or Rc. Size bar: 25 μm. (**B**) Expression profiles of genes related to autophagy. Expression levels of autophagy-associated genes were also higher at 24 h than at 48 h after treatment. Fold-change in gene expression was normalized to *nfgapdh* as an internal control gene. MF (IC_90_) was included as the positive control. NC, negative control. **** *p* < 0.0001, *** *p* < 0.001, and * *p* < 0.1 compared to NC. ^####^ *p* < 0.0001, ^###^ *p* < 0.001, ^##^ *p* < 0.01, and ^#^ *p* < 0.1 compared to MF.

Blue fluorescence of autophagosomes was detected in *N. fowleri* trophozoites treated with Rb3 or Rc, suggesting that these two ginsenosides induced autophagy in amoebae (Figure 6A). Interestingly, the fluorescence intensity was stronger in amoebae at 24 h than at 48 h post-treatment. Expression levels of autophagy-associated genes such as *nfatg2*, *nfatg5*, *nfatg8*, and *nfatg12* were also higher at 24 h than those at 48 h after treatment (Figure 6B). In particular, *nfatg8* and *nfatg12* showed profound upregulation.

## 3. Discussion

Ginsenosides, bioactive substances derived from *Panax* ginseng, have demonstrated a broad range of pharmacological effects, such as anti-inflammatory, anticancer, antioxidant, anti-diabetic, and neuroprotective effects [34,35,36,37,38,39,40,41]. Antimicrobial effects of ginsenosides have also been studied [26,32,33]. However, their potential antiprotozoal activity against pathogenic protozoans has remained largely unexplored. As an extension of our research to discover novel natural compounds as potential PAM drugs or alternatives, we explored anti-*N. fowleri* activities of 11 ginsenosides from *Panax* ginseng and found that ginsenosides Rb3 and Rc exhibited promising and selective anti-*N. fowleri* activities without showing significant cytotoxicity against C6 glial cells, resulting in low values of IC_50_ and high values of CC_50_ and SI, surpassing those of MF, a drug of choice for PAM. IC_50_ and IC_90_ values of Rb3 were lower than those of Rc, suggesting that Rb3 possessed stronger anti-*N. fowleri* activity than Rc. Nevertheless, overall values imply selective anti-*N. fowleri* activity of Rc was within acceptable ranges. Different anti-*N. fowleri* activity and C6 glial cell cytotoxicity of the 11 tested ginsenosides suggest a possible structure–activity relationship. Ginsenosides are primarily classified into three major types based on the structure of aglycone backbones: protopanaxadiol (PPD) type, protopanaxatriol (PPT) type, and oleanolic acid (OA) type [42]. PPD and PPT differ in the positions of sugar attachment on the dammarane core, whereas OA possesses a distinct sapogenin backbone. PPD types (CK, Rb3, Rc, Rg3, and Rh2) exhibited anti-*N. fowleri* activity, but PPT types (Rg1, Rg2, Rh1, Re, and F1) and OA type (Ro) did not, suggesting that the structural difference might be associated with anti-amoeba activity. Highly glycosylated ginsenosides generally exhibit lower membrane permeability than less glycosylated or deglycosylated forms [43,44]. Among the PPD type ginsenosides applied in this study, Rb3 and Rc are more glycosylated forms, whereas CK, Rg3, and Rh2 are less glycosylated derivatives, suggesting that differences in membrane permeability might also affect the selective anti-*N. fowleri* activity of Rb3 and Rc with less cytotoxicity. However, further comprehensive studies to clarify the relationship between structure and selective anti-*N. fowleri* activity of ginsenosides is warranted.

**Figure 7:**
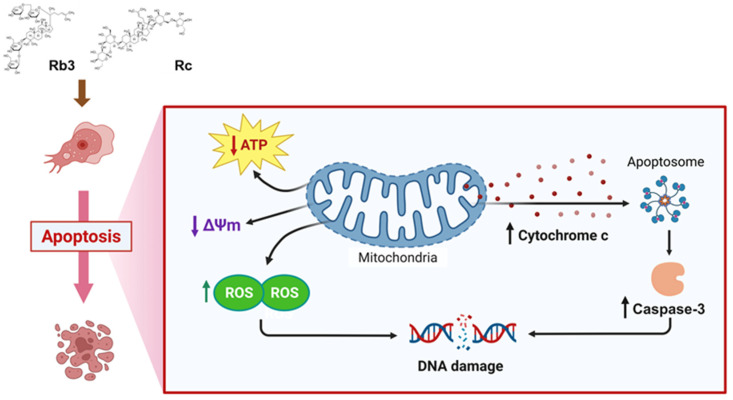
Summary of potential anti-amoebic mechanism of ginsenosides Rb3 and Rc in *N. fowleri* trophozoites. Rb3 and Rc induced apoptosis-like PCD by increasing intracellular ROS levels, which might have subsequently caused mitochondrial dysfunction, increased caspase-3 activity, and promoted DNA fragmentation, eventually leading to amoeba death.

Apoptosis, a form of PCDs, is a complicated but strictly regulated process that plays an essential role in maintaining cellular homeostasis and eliminating non-functional or damaged cells [23]. It is typically characterized by a series of specific morphological changes, such as cell blebbing and shrinkage, along with energy-dependent biochemical and physiological events, including chromatin condensation, DNA fragmentation, loss of mitochondrial membrane potential, and externalization of phosphatidylserine [23]. Although classical apoptotic pathways in protozoa may differ from those in higher eukaryotes, apoptosis and apoptosis-like mechanisms have also been documented in unicellular protozoan parasites, suggesting that they share functionally analogous pathways to mediate the regulation event with multicellular organisms [45]. Diverse natural and synthetic compounds have been investigated for PAM drug candidates that induce apoptosis-like PCD in *N. fowleri* [17,20,24,46,47,48,49,50]. The results of the present study aligned with previous findings, demonstrating that *N. fowleri* trophozoites underwent similar apoptosis-like PCD upon exposure to Rb3 or Rc. Characteristic morphological changes, such as size reduction and shape deformation indicative of PCD, were observed in *N. fowleri* trophozoites treated with Rb3 or Rc. Although ultrastructural morphological changes associated with PCD in the amoeba were not investigated in this study, results of complementary assays, including strong apoptotic signals, DNA fragmentation, and increased ROS production identified in *N. fowleri* trophozoites upon treatment with Rb3 or Rc, support the occurrence of PCD in the amoeba. Signals indicating mitochondrial damage, such as depolarization of mitochondrial membrane potential, significant reduction in ATP production, and enhanced expression of mitochondrial genes associated with mitochondrial dysfunction, were also found in Rb3- or Rc-treated amoebae. Damaged mitochondria are known to release cytochrome c and other factors that can activate the PCD cascade events and contribute to cell death [51]. Caspase-3, a well-known executioner caspase in apoptosis pathways, plays a central role in PCD. Its activation is a hallmark of PCD [52]. Enhanced caspase-3 activity in Rb3- or Rc-treated *N. fowleri* trophozoites also supports the notion that these two ginsenosides can evoke apoptosis-like PCD in the amoebae, facilitating amoebae death. Taken together, Rb3 and Rc are likely to exert anti-*N. fowleri* activities by inducing a cellular event associated with intrinsic apoptosis-like PCD in the amoeba (Figure 7). However, we were not able to investigate the in-depth molecular pathway and associated molecules involved in this event primarily due to the highly limited information on molecules involved in PCD in *N. fowleri* and a lack of essential materials, such as specific antibodies and proteins, to investigate pathways in the amoeba. Therefore, further comprehensive studies are necessary to elucidate a systematic path of PCD in *N. fowleri* induced by Rb3 and Rc.

Our results also suggest that Rb3 and Rc can induce autophagy activation in *N. fowleri* trophozoites. Autophagy is a unique cellular process that degrades and recycles damaged organelles and macromolecules. While autophagy often serves as a survival strategy under stressed conditions, excessive or dysregulated autophagy can contribute to cell death [53]. Increases in autophagosome formation and expressions of autophagy-associated genes were detected in *N. fowleri* upon treatment with Rb3 or Rc, suggesting that these two ginsenosides could trigger autophagy formation in the amoeba. The potential role of Atg8 in apoptosis-like PCD and autophagy formation in *N. gruberi* and *N. fowleri* has been reported [48]. However, the biological roles of the other Atgs, including Atg12, are not yet clearly understood in *N. fowleri*. The biological functions of Atg have been largely investigated in *Acanthamoeba*. In *Acanthamoeba*, Atg5, Atg12, and Atg16 play crucial roles in the autophagosome formation, particularly during encystation [54,55]. Enhanced expressions of Atg genes have been identified in *Acanthamoeba* under harsh stress conditions that cause death, but no induction of cyst formation is observed [16,56], suggesting that the amoeba undergoes encystation processes under these conditions to protect itself; however, it dies before mature cyst formation occurs. Similar phenomena were also identified in *N. fowleri* treated with compounds that induce amoeba death [17,24], suggesting that Atgs of *N. fowleri* might also play roles in autophagosome formation, thereby facilitating the autophagy pathway; however, this requires further confirmation. Interestingly, signals of autophagy were stronger in the early phase of treatment (24 h) than in the late phase (48 h), which did not align with the time-dependent increases in apoptosis signals identified. This could be explained by the hypothesis that Rb3 and Rc might have acted as stress factors, inducing autophagy formation in the amoeba and serving an initial protective function, but the forces gradually decreased as the amoeba died. Alternatively, the excessively formed autophagy in the early phase might have paradoxically accelerated amoeba death. Further studies are warranted to clarify the underlying mechanisms and relationships between autophagy and PCD in Rb3- or Rc-treated *N. fowleri*.

The pharmacological benefits and modes of action of Rb3 and Rc in preventing and treating human diseases have been extensively studied [34,57,58,59]. The safety of ginsenosides has also been confirmed in previous studies, emphasizing the potential of these ginsenosides as attractive PAM drug candidates. However, several challenges still remain. One major hurdle in developing PAM drugs is blood–brain barrier (BBB) permeability. Given that the brain is the infection loci of *N. fowleri*, PAM drugs should pass the BBB to reach the infected foci. It has been reported that some ginsenosides, such as Rd and F1, can easily cross the BBB and exert neuroprotective effects in certain models [60,61], while other ginsenosides, such as Rg1, display limited BBB permeability unless they are modified or delivered via carriers [62]. BBB permeabilities of Rb3 and Rc have not yet been clearly investigated. Therefore, more comprehensive studies on the brain-delivery potential of these ginsenosides, as well as further in vivo therapeutic efficacy studies using animal models, are needed. Ginseng contains over 200 ginsenosides with diverse pharmacological functions [63]. In this study, we explored only a small number of ginsenosides, which was a limitation of this study. Further exploration is needed to determine the anti-*N. fowleri* activity of other ginsenosides and their potential as PAM drug candidates.

## 4. Materials and Methods

### 4.1. Chemicals

Eleven ginsenosides, including CK, Rb3, Rc, Rg1, Rg2, Rg3, Rh1, Rh2, Re, Ro, and F1, and miltefosine (MF; purity > 98%) were purchased from Sigma-Aldrich (St. Louis, MO, USA). These ginsenosides and MF were dissolved in a 25 mM stock solution in dimethyl sulfoxide (DMSO; Sigma-Aldrich) and used in this study.

### 4.2. Naegleria fowleri and C6 Glial Cells

*N. fowleri* (ATCC 30215, Carter NF69 strain; American Type Culture Collection, Manassas, VA, USA) was axenically cultured in Nelson’s medium, containing 1% penicillin/streptomycin (P/S; Gibco, Grand Island, NY, USA) and 5% fetal bovine serum (FBS; Gibco). The C6 rat glial cells (C6 cells; ATCC CCL-107) were cultured in Dulbecco’s Modified Eagle’s Medium (DMEM; Gibco), supplemented with 1% P/S (Gibco) and 10% FBS (Gibco). *N. fowleri* and C6 glial cells were incubated at 37 °C in 5% CO_2_ incubator.

### 4.3. Anti-N. fowleri Activity Assay

Anti-*N. fowleri* activity of each ginsenoside was analyzed in a 96-well microplate format [17]. Briefly, serial dilutions of each ginsenoside, ranging from 0 to 200 μM, were treated with freshly cultured *N. fowleri* trophozoites (5 × 10^4^ amoebae/well) in 96-well microplates (Thermo Fisher Scientific, Waltham, MA, USA) and incubated at 37 °C for 48 h. Morphological changes in amoebae were observed using an EVOS XL Core microscope (Thermo Fisher Scientific). CellTiter-Blue^®^ Cell Viability Assay (Promega, Madison, WI, USA) was used to assess cell viability. Inhibitory concentration of 50% (IC_50_) and 90% (IC_90_) was calculated using Prism 10.2.3 (GraphPad, San Diego, CA, USA).

### 4.4. Cytotoxicity Assay for C6 Glial Cells

C6 glial cells were added into 96-well microplates (Thermo Fisher Scientific) at a density of 2 × 10^4^ cells/well. Serial dilutions of each ginsenoside (0−200 μM) were treated to C6 glial cells, and the cells were incubated at 37 °C for 48 h. Morphological changes and cell viability were determined using the same methods described above. Cytotoxic concentration of 50% (CC_50_) was determined using Prism 10.2.3 (GraphPad). The sensitivity index (SI) was calculated as the ratio of CC_50_ to IC_50_.

### 4.5. Apoptosis/Necrosis Assay

An apoptosis/necrosis assay was conducted using an Apoptosis/Necrosis Detection Kit (Abcam, Cambridge, UK). *N. fowleri* trophozoites (5 × 10^4^ amoebae) were seeded into each well of a 96-well black/clear bottom plate (Thermo Fisher Scientific). After allowing amoebae to adhere to the bottom of wells, Rb3 (IC_90_) or Rc (IC_90_) was added to the amoebae, followed by incubation at 37 °C for 24 or 48 h. Amoebae treated with 0.1% DMSO without Rb3 and Rc were used as negative controls. MF (IC_90_)-treated amoebae were applied as positive controls. At each time point, CytoCalcein, Green Apopxin, and 7-Aminoactinomycin D (7-AAD) were used to stain amoebae, which were then incubated at 37 °C in the dark for 1 h. Amoebae were rinsed twice with FBS-free Nelson’s media, and fluorescence signals were visualized using an EVOS M5000 fluorescence microscope (Thermo Fisher Scientific). Three fluorescence channels were subjected: the DAPI channel (Ex/Em = 405/450 nm) for CytoCalcein Violet 450, the GFP channel (Ex/Em = 490/525 nm) for Green Apopxin, and the Texas Red channel (Ex/Em = 550/650 nm) for 7-AAD. The apoptotic effects of Rb3 and Rc against C6 glial cells were also analyzed with the same method.

### 4.6. Terminal Deoxynucleotidyl Transferase dUTP Nick End Labeling (TUNEL) Assay

TUNEL assay was performed on 12-well microplates (Thermo Fisher Scientific) using an in situ Direct DNA Fragmentation Assay Kit (Abcam). *N. fowleri* trophozoites (3 × 10^5^ amoebae/well) seeded into plates were treated with Rb3 (IC_90_) or Rc (IC_90_) and incubated at 37 °C for 24 or 48 h, respectively. Amoebae were collected, fixed with 3.7% formaldehyde, and rinsed with phosphate-buffered saline (PBS) [17]. Cells were harvested and resuspended in 1 mL of 70% ethanol, then placed on ice for 30 min. Amoebae were double-stained with TUNEL and propidium iodide (PI) following the manufacturer’s instructions. Fluorescein-labeled DNA was visualized using a fluorescence microscope (EVOS M5000, Thermo Fisher Scientific) in the GFP channel for TUNEL and the Texas Red channel for PI. Amoebae treated with 0.1% DMSO and MF (IC_90_) in Nelson’s medium were used as negative and positive controls, respectively.

### 4.7. Intracellular Reactive Oxygen Species (ROS) Assay

Changes in intracellular ROS levels in *N. fowleri* trophozoites upon treatment of Rb3 or Rc were assessed using a DCFDA/H2DCFDA–Cellular ROS Assay Kit (Abcam). *N. fowleri* trophozoites (5 × 10^4^ amoebae/well) seeded into a 96-well black/clear bottom microplate (Thermo Fisher Scientific) were treated with Rb3 (IC_90_) or Rc (IC_90_) and incubated at 37 °C for 24 or 48 h. At the indicated time points, amoebae were incubated with a 20 μM 2× DCFDA solution and maintained at 37 °C in the dark for 45 min. Fluorescence intensity was subsequently measured using a VICTOR Nivo Multimode microplate reader (PerkinElmer, Waltham, MA, USA) at Ex/Em wavelength of 480/530 nm. The green fluorescence signal indicating the generation of ROS was qualitatively assessed using an EVOS M5000 fluorescence microscope (Thermo Fisher Scientific) in the GFP channel. Amoebae treated with 0.1% DMSO and MF (IC_90_) in Nelson’s medium were used as negative and positive controls, respectively. Effects of Rb3 and Rc on the ROS generation in C6 glial cells were also analyzed. Cells (2 × 10^4^ cells/well) seeded into a 96-well black/clear bottom microplate (Thermo Fisher Scientific) were treated with Rb3 (IC_90_) or Rc (IC_90_) and incubated at 37 °C in the dark for 24 or 48 h, respectively. ROS levels were measured with the same protocols.

### 4.8. Mitochondrial Membrane Potential Assay

JC-1 Mitochondrial Membrane Potential Assay Kit (Abcam) was used to analyze the change in the electrochemical gradient across the mitochondrial membrane of *N. fowleri* upon treatment with Rb3 or Rc. Amoeba trophozoites (5 × 10^4^ amoebae) seeded into a 96-well black/clear bottom microplate (Thermo Fisher Scientific) were treated with Rb3 (IC_90_) or Rc (IC_90_) and incubated at 37 °C for 24 or 48 h. At the indicated time point, 50 μL of 2× JC-1 solution (10 μM) was added to the amoebae, and the plate was further incubated in the dark at 37 °C for 45 min. Green and red fluorescence signals were visualized using an EVOS M5000 fluorescence microscope (Thermo Fisher Scientific) with the GFP channel for JC-1 monomer and the Texas Red channel for JC-1 aggregate, respectively. Amoebae treated with 0.1% DMSO were used as a negative control, while amoebae treated with MF (IC_90_) were used as a positive control.

### 4.9. Measurement of ATP Levels

Changes in ATP levels in *N. fowleri* trophozoites treated with Rb3 or Rc were analyzed using a Luminescent ATP Detection Assay Kit (Abcam). *N. fowleri* trophozoites (5 × 10^4^ amoebae/well) cultured in 96-well white/clear bottom microplates (Thermo Fisher Scientific) were treated with Rb3 (IC_90_) or Rc (IC_90_). After incubation for 24 or 48 h, detergent and substrate solutions were added to each well. Plates were kept in the dark for 10 min, and luminescence was then measured using a VICTOR Nivo Multimode Microplate Reader (PerkinElmer). The amoeba trophozoites treated with 0.1% DMSO were used as a negative control, while amoebae treated with MF (IC_90_) were used as a positive control.

### 4.10. Caspase-3 Activity Assay

To further analyze the relationship between apoptosis and *N. fowleri* death caused by Rb3 or Rc, caspase-3 activity in amoebae treated with Rb3 or Rc was assessed using a NucView^®^ 488 Caspase-3 Assay Kit (Biotium, Fremont, CA, USA). *N. fowleri* trophozoites (5 × 10^4^ amoebae/well) seeded into a black/clear bottom 96-well microplate (Thermo Fisher Scientific) were treated with Rb3 (IC_90_) or Rc (IC_90_) for 24 or 48 h. After adding a 2× solution of NucView^®^ 488 substrate (5 μM) to each well, the plate was incubated in the dark for 30 min, and fluorescence signals were observed in the GFP channel using an EVOS M5000 fluorescence microscope (Thermo Fisher Scientific). Fluorescence intensity was also measured at Ex/Em wavelengths of 480/530 nm using a VICTOR Nivo Multimode Microplate Reader (PerkinElmer). Amoebae treated with 0.1% DMSO and MF (IC_90_) were used as a negative control and a positive control, respectively.

### 4.11. Autophagy Assay

Autophagy induction in *N. fowleri* trophozoites by Rb3 or Rc treatment was evaluated using an Autophagy Assay Kit (Sigma-Aldrich). Amoeba trophozoites (5 × 10^4^ amoe-bae/well) seeded into a black/clear bottom 96-well microplate (Thermo Fisher Scientific) were treated with Rb3 (IC_90_) or Rc (IC_90_) for 24 or 48 h. After an autophagosome detection reagent was added to each well, the plate was incubated in the dark for 1 h. Blue fluorescence signals, implying autophagosome formation, were observed using the EVOS M5000 fluorescence microscope (Thermo Fisher Scientific) in the DAPI channel. Amoebae treated with 0.1% DMSO and MF (IC_90_) were used as a negative control and a positive control, respectively.

### 4.12. Quantitative Reverse Transcription Polymerase Chain Reaction (qRT-PCR)

To assess transcriptional changes in apoptosis- and autophagy-related genes in *N. fowleri* upon treatment by Rb3 or Rc, amoeba trophozoites (2 × 10^6^ amoebae/well) in a 6-well plate were treated with Rb3 (IC_90_) or Rc (IC_90_). After incubation at 37 °C for 24 or 48 h, amoebae were harvested and rinsed with PBS. RNA from each sample was purified using a RNeasy^®^ Plus Mini Kit (Qiagen, Hilden, Germany). RNA concentration of each sample was measured using a Nanodrop spectrophotometer (DeNovix DS-11; Wilmington, DE, USA) and then equalized. RNA was transcribed into complementary DNA (cDNA) using the RNA to cDNA EcoDry Premix (Clontech, Mountain View, CA, USA). The cDNA was then used for qPCR with a QuantStudio 1 Real-Time PCR System (Thermo Fisher Scientific). Specific primers for *N. fowleri*, including apoptosis-including factor 2-like (*nfaif2*), cytochrome c1 (*nfcytc1*), programmed cell death protein 6 (*nfpcd6*), and autophagy-related genes (*nfatg2*, *nfatg5*, *nfatg8*, and *nfatg12*), were used with the protocols described previously [17]. *N. fowleri* glyceraldehyde-3-phosphate dehydrogenase (*nfgapdh*) was used as an internal control [64].

### 4.13. Statistical Analysis

All experiments were performed in three independent experiments, each in duplicate. All results were represented as mean and standard deviation (SD) from three independent assays. Statistical significance was analyzed by a one-way analysis of variance (ANOVA) with Dunnett’s post hoc test using Prism 10.2.3 (GraphPad). A *p*-value less than 0.01 is considered statistically significant.

## 5. Conclusions

This study provided the first evidence that ginsenosides Rb3 and Rc possess promising anti-amoebic activities against *N. fowleri* trophozoites without showing significant cytotoxicity to mammalian cells. Ginsenosides Rb3 and Rc are likely to induce apoptosis-like PCD in *N. fowleri* trophozoites, characterized by a series of cellular mechanisms, such as ROS generation, mitochondrial dysfunction, and caspase-3 activation. These findings offer a strong foundation for further exploration of ginsenosides Rb3 and Rc as potential adjunctive or alternative therapeutic candidates for PAM. Future research is warranted to elucidate the precise molecular mechanisms underlying Rb3- and Rc-induced apoptosis-like PCD in *N. fowleri* trophozoites and assess in vivo therapeutic efficacies and BBB permeabilities of Rb3 and Rc.

## Figures and Tables

**Table 1:** Anti-amoebic activity of compounds against *N. fowleri*.

Compounds	*N. fowleri*		C6	SI
	IC_50_ ± SD (μM)	IC_90_ ± SD (μM)	CC_50_ ± SD (μM)	
CK	60.16 ± 3.15	96.38 ± 5.21	69.60 ± 0.05	1.17
Rb3	94.71 ± 1.63	170.96 ± 0.71	963.23 ± 32.23	10.17
Rc	126.99 ± 1.88	229.23 ± 0.31	>1000	>7.87
Rg1	>200	>200	>200	-
Rg2	>200	>200	>200	-
Rg3	83.00 ± 3.89	178.12 ± 1.27	304.49 ± 0.14	3.67
Rh1	>200	>200	>200	-
Rh2	47.19 ± 0.39	86.01 ± 4.48	69.69 ± 0.09	1.48
Re	>200	>200	>200	-
Ro	>200	>200	>200	-
F1	>200	>200	>200	-
MF	92.25 ± 0.73	122.22 ± 1.37	140.42 ± 1.22	1.52

IC, inhibitory concentration; CC, cytotoxicity concentration; SI, selectivity index (CC_50_/IC_50_).

## Data Availability

The original contributions presented in this study are included in the article. Further inquiries can be directed to the corresponding author.

## Abbreviations

7-AAD, 7-aminoactinomycin D; AmpB, amphotericin B; ANOVA, analysis of variance; atg, autophagy-related gene; CC_50_, cytotoxicity concentration 50; CNS, central nervous system; DAPI, 4′,6-diamidino-2-phenylindole; DCFDA, 2′,7′-dichlorofluorescein diacetate; DMEM, Dulbecco’s modified Eagle’s medium; DMSO, dimethyl sulfoxide; FBS, fetal bovine serum; gapdh, glyceraldehyde-3-phosphate dehydrogenase; IC_50_, inhibitory concentration 50; IC_90_, inhibitory concentration 90; MF, miltefosine; PAM, primary amoebic meningoencephalitis; PBS, phosphate-buffered saline; PCD, programmed cell death; PI, propidium iodide; ROS, reactive oxygen species; P/S, penicillin/streptomycin; RT, room temperature; qRT-PCR, quantitative reverse transcription polymerase chain reaction; SD, standard deviation; SI, sensitivity index; TUNEL, terminal deoxynucleotidyl transferase dUTP end-labeling.

## References

[1] Grace E., Asbill S., Virga K. (2015). *Naegleria fowleri*: Pathogenesis, diagnosis, and treatment options. Antimicrob. Agents Chemother..

[2] De Jonckheere J.F. (2011). Origin and evolution of the worldwide distributed pathogenic amoeboflagellate *Naegleria fowleri*. Infect. Genet. Evol..

[3] Jarolim K.L., McCosh J.K., Howard M.J., John D.T. (2000). A light microscopy study of the migration of *Naegleria fowleri* from the nasal submucosa to the central nervous system during the early stage of primary amebic meningoencephalitis in mice. J. Parasitol..

[4] Marciano-Cabral F., Cabral G.A. (2007). The immune response to *Naegleria fowleri* amebae and pathogenesis of infection. FEMS Immunol. Med. Microbiol..

[5] Lê H.G., Kang J.M., Võ T.C., Na B.K. (2022). *Naegleria fowleri* Cathepsin B induces a pro-inflammatory immune response in BV-2 microglial cells via NF-κB and AP-1 dependent-MAPK signaling pathway. Int. J. Mol. Sci..

[6] Lê H.G., Kang J.M., Võ T.C., Yoo W.G., Na B.K. (2023). *Naegleria fowleri* extracellular vesicles induce proinflammatory immune responses in BV-2 microglial cells. Int. J. Mol. Sci..

[7] Alanazi A., Younas S., Ejaz H., Alruwaili M., Alruwaili Y., Mazhari B.B.Z., Atif M., Junaid K. (2025). Advancing the understanding of *Naegleria fowleri*: Global epidemiology, phylogenetic analysis, and strategies to combat a deadly pathogen. J. Infect. Public Health.

[8] Gharpure R., Bliton J., Goodman A., Ali I.K.M., Yoder J., Cope J.R. (2020). Epidemiology and clinical characteristics of primary amebic meningoencephalitis caused by *Naegleria fowleri*: A global review. Clin. Infect. Dis..

[9] Güémez A., García E. (2021). Primary amoebic meningoencephalitis by *Naegleria fowleri*: Pathogenesis and treatments. Biomolecules.

[10] Leal dos Santos D., Chaúque B.J.M., Virginio V.G., Cossa V.C., Pettan-Brewer C., Schrekker H.S., Rott M.B. (2022). Occurrence of *Naegleria fowleri* and their implication for health—A look under the One Health approaches. Int. J. Hyg. Environ. Health.

[11] Cope J.R., Conrad D.A., Cohen N., Cotilla M., Dasilva A., Jackson J., Visvesvara G.S. (2015). Use of the novel therapeutic agent miltefosine for the treatment of primary amebic meningoencephalitis: Report of 1 fatal and 1 surviving case. Clin. Infect. Dis..

[12] Stowe R.C., Pehlivan D., Friederich K.E., Lopez M.A., DiCarlo S.M., Boerwinkle V.L. (2017). Primary amebic meningoencephalitis in children: A report of two fatal cases and review of the literature. Pediatr. Neurol..

[13] Haston J.C., Cope J.R. (2023). Amebic encephalitis and meningoencephalitis: An update on epidemiology, diagnostic methods, and treatment. Curr. Opin. Infect. Dis..

[14] Chaúque B.J.M., da Silva T.C.B., Rott E.B., Rott F.B., Leite A.P.M.C., Benitez G.B., Neuana N.F., Goldim J.R., Rott M.B., Zanette R.A. (2025). Effectiveness of phytoproducts against pathogenic free-living amoebae—A scoping and critical review paving the way toward plant-based pharmaceuticals. Fitoterapia.

[15] Lê H.G., Kim W., Kang J.M., Võ T.C., Yoo W.G., Cheong H., Na B.-K. (2024). The anti-amoebic activity of *Pinus densiflora* leaf extract against the brain-eating amoeba *Naegleria fowleri*. Parasites Hosts Dis..

[16] Lê H.G., Kang J.M., Võ T.C., Yoo W.G., Hong Y., Na B.K. (2024). (−)-Epicatechin reveals amoebicidal activity against *Acanthamoeba castellanii* by activating the programmed cell death pathway. Phytomedicine.

[17] Lê H.G., Kang J.M., Võ T.C., Na B.K. (2023). Kaempferol induces programmed cell death in *Naegleria fowleri*. Phytomedicine.

[18] Lê H.G., Võ T.C., Kang J.M., Nguyễn T.H., Hwang B.S., Oh Y.T., Na B.-K. (2023). Antiamoebic activities of flavonoids against pathogenic free-living amoebae, *Naegleria fowleri* and *Acanthamoeba* species. Parasites Hosts Dis..

[19] Rajendran K., Ahmed U., Meunier A.C., Shaikh M.F., Siddiqui R., Anwar A. (2023). Natural terpenes inhibit the cytopathogenicity of *Naegleria fowleri* causing primary amoebic meningoencephalitis in the human cell line model. ACS Chem. Neurosci..

[20] Rizo-Liendo A., Sifaoui I., Arberas-Jiménez I., Reyes-Batlle M., Piñero J.E., Lorenzo-Morales J. (2020). Fluvastatin and atorvastatin induce programmed cell death in the brain eating amoeba *Naegleria fowleri*. Biomed. Pharmacother..

[21] Zeouk I., Sifaoui I., Rizo-Liendo A., Arberas-Jiménez I., Reyes-Batlle M., Bazzocchi I., Bekhti K., Piñero J.E., Jiménez I.A. (2021). Exploring the anti-infective value of inuloxin A isolated from *Inula viscosa* against the brain-eating amoeba (*Naegleria fowleri*) by activation of programmed cell death. ACS Chem. Neurosci..

[22] Belofsky G., Carreno R., Goswick S., John D. (2006). Activity of isoflavans of *Dalea aurea* (Fabaceae) against the opportunistic ameba *Naegleria fowleri*. Planta Med..

[23] Yuan J., Ofengeim D. (2024). A guide to cell death pathways. Nat. Rev. Mol. Cell Biol..

[24] Lê H.G., Hwang B.S., Choi J.S., Jeong Y.T., Võ T.C., Cho M., Hong Y., Kim J.H., Oh Y.T., Na B.-K. (2025). A xanthone O-glucoside isolated from *Iris setosa* Pall. ex Link exhibits promising anti-amoebic activity against the brain-eating amoeba *Naegleria fowleri*. Phytomedicine.

[25] Clardy J., Walsh C. (2004). Lessons from natural molecules. Nature.

[26] Ratan Z.A., Haidere M.F., Hong Y.H., Park S.H., Lee J.O., Lee J., Cho J.Y. (2021). Pharmacological potential of ginseng and its major component ginsenosides. J. Ginseng Res..

[27] Sng K.S., Li G., Zhou L., Song Y., Chen X., Wang Y., Yao M., Cui X. (2022). Ginseng extract and ginsenosides improve neurological function and promote antioxidant effects in rats with spinal cord injury: A meta-analysis and systematic review. J. Ginseng Res..

[28] Liu Y., Zhang H., Dai X., Zhu R., Chen B., Xia B., Ye Z., Zhao D., Gao S., Orekhov A.N. (2021). A comprehensive review on the phytochemistry, pharmacokinetics, and antidiabetic effect of Ginseng. Phytomedicine.

[29] Niu J., Zhu G., Zhang J. (2025). Ginseng in delaying brain aging: Progress and Perspectives. Phytomedicine.

[30] Niu Z., Liu Y., Shen R., Jiang X., Wang Y., He Z., Li J., Hu Y., Zhang J., Jiang Y. (2024). Ginsenosides from *Panax* ginseng as potential therapeutic candidates for the treatment of inflammatory bowel disease. Phytomedicine.

[31] Lee C.H., Kim J.H. (2014). A review on the medicinal potentials of ginseng and ginsenosides on cardiovascular diseases. J. Ginseng Res..

[32] Im K., Kim J., Min H. (2016). Ginseng, the natural effectual antiviral: Protective effects of Korean red ginseng against viral infection. J. Ginseng Res..

[33] Kachur K., Suntres Z.E. (2015). The antimicrobial properties of ginseng and ginseng extracts. Expert Rev. Anti-Infect. Ther..

[34] Sun M., Ji Y., Li Z., Chen R., Zhou S., Liu C., Du M. (2020). Ginsenoside Rb3 inhibits pro-inflammatory cytokines via MAPK/AKT/NF-κB pathways and attenuates rat alveolar bone resorption in response to *Porphyromonas gingivalis* LPS. Molecules.

[35] He X.L., Xu X.H., Shi J.J., Huang M., Wang Y., Chen X., Lu J. (2021). Anticancer effects of ginsenoside Rh2: A systematic review. Curr. Mol. Pharmacol..

[36] Paik S., Song G.Y., Jo E.K. (2023). Ginsenosides for therapeutically targeting inflammation through modulation of oxidative stress. Int. Immunopharmacol..

[37] Gong L., Yin J., Zhang Y., Huang R., Lou Y., Jiang H., Sun L., Jia J., Zeng X. (2022). Neuroprotective mechanisms of ginsenoside Rb1 in central nervous system diseases. Front. Pharmacol..

[38] Han Y., Wang T., Li C., Wang Z., Zhao Y., He J., Fu L., Han B. (2021). Ginsenoside Rg3 exerts a neuroprotective effect in rotenone-induced Parkinson’s disease mice via its anti-oxidative properties. Eur. J. Pharmacol..

[39] Xie J.T., Shao Z.H., Vanden Hoek T.L., Chang W.T., Li J., Mehendale S., Wang C., Hsu C., Becker L.B., Yin J. (2006). Antioxidant effects of ginsenoside Re in cardiomyocytes. Eur. J. Pharmacol..

[40] Chen W., Balan P., Popovich D.G. (2019). Review of ginseng anti-diabetic studies. Molecules.

[41] Yang Y., Nan Y., Du Y., Liu W., Ning N., Chen G., Gu Q., Yuan L. (2024). Ginsenosides in cancer: Proliferation, metastasis, and drug resistance. Biomed. Pharmacother..

[42] Shi Z.Y., Zeng J.Z., Tsai Wong A.S. (2019). Chemical structures and pharmacological profiles of ginseng saponins. Molecules.

[43] Chu L.L., Huy N.Q., Tung N.H. (2023). Microorganisms for ginsenosides biosynthesis: Recent Progress, Challenges, and Perspectives. Molecules.

[44] Ku S. (2016). Finding and producing probiotic glycosylases for the biocatalysis of ginsenosides: A mini review. Molecules.

[45] Kaczanowski S., Sajid M., Reece S.E. (2011). Evolution of apoptosis-like programmed cell death in unicellular protozoan parasites. Parasit. Vectors.

[46] Arberas-Jiménez I., Rodríguez-Expósito R.L., San Nicolás-Hernández D., Chao-Pellicer J., Sifaoui I., Díaz-Marrero A.R., Fernández J.J., Piñero J.E., Lorenzo-Morales J. (2023). Marine meroterpenoids isolated from *Gongolaria* abies-marina induce programmed cell death in *Naegleria fowleri*. Pharmaceuticals.

[47] Cárdenas-Zúñiga R., Silva-Olivares A., Villalba-Magdaleno J.D.A., Sánchez-Monroy V., Serrano-Luna J., Shibayama M. (2017). Amphotericin B induces apoptosis-like programmed cell death in *Naegleria fowleri* and *Naegleria gruberi*. Microbiology.

[48] Arberas-Jiménez I., García-Davis S., Rizo-Liendo A., Sifaoui I., Morales E.Q., Piñero J.E., Lorenzo-Morales J., Díaz-Marrero A.R., Fernández J.J. (2022). Cyclolauranes as plausible chemical scaffold against *Naegleria fowleri*. Biomed. Pharmacother..

[49] Chao-Pellicer J., Arberas-Jiménez I., Delgado-Hernández S., Sifaoui I., Tejedor D., García-Tellado F., Piñero J.E., Lorenzo-Morales J. (2023). Cyanomethyl vinyl ethers against *Naegleria fowleri*. ACS Chem. Neurosci..

[50] Arberas-Jiménez I., Rizo-Liendo A., Nocchi N., Sifaoui I., Chao-Pellicer J., Souto M.L., Suárez-Gómez B., Díaz-Marrero A.R., Fernández J.J., Piñero J.E. (2022). Sesquiterpene lactones as potential therapeutic agents against *Naegleria fowleri*. Biomed. Pharmacother..

[51] Goldstein J.C., Waterhouse N.J., Juin P., Evan G.I., Green D.R. (2000). The coordinate release of cytochrome c during apoptosis is rapid complete and kinetically invariant. Nat. Cell Biol..

[52] Porter A.G., Jänicke R.U. (1999). Emerging roles of caspase-3 in apoptosis. Cell Death Differ..

[53] Liu S.Z., Yao S.J., Yang H., Liu S.J., Wang Y.J. (2023). Autophagy: Regulator of cell death. Cell Death Dis..

[54] Kim S.-H., Moon E.-K., Hong Y., Chung D.-I., Kong H.-H. (2015). Autophagy protein 12 plays an essential role in *Acanthamoeba* encystation. Exp. Parasitol..

[55] Song S.M., Han B.I., Moon E.K., Lee Y.R., Yu H.S., Jha B.K., Danne D.-B.S., Kong H., Chung D., Hong Y. (2012). Autophagy protein 16-mediated autophagy is required for the encystation of *Acanthamoeba castellanii*. Mol. Biochem. Parasitol..

[56] Boonhok R., Sangkanu S., Norouzi R., Siyadatpanah A., Mirzaei F., Mitsuwan W., Charong N., Wisessombat S., Pereira M.d.L., Rahmatullah M. (2021). Amoebicidal activity of Cassia angustifolia extract and its effect on *Acanthamoeba* triangularis autophagy-related gene expression at the transcriptional level. Parasitology.

[57] Oh H., Cho W., Park S.Y., Abd El-Aty A.M., Jeong J.H., Jung T.W. (2023). Ginsenoside Rb3 ameliorates podocyte injury under hyperlipidemic conditions via PPARδ- or SIRT6-mediated suppression of inflammation and oxidative stress. J. Ginseng Res..

[58] Chen X., Yan X., Jing C., Fu B., Jin W., Zhang S., Wang M., Liu F., Sun L. (2025). Ginsenoside Rc maintains sleep rhythm homeostasis by alleviating oxidative stress. Phytomedicine.

[59] Kim A., Park S.M., Kim N.S., Park M., Cha S. (2024). Ginsenoside Rc prevents dexamethasone-induced muscle atrophy and enhances muscle strength and motor function. J. Ginseng Res..

[60] Ye R., Kong X., Yang Q., Zhang Y., Han J., Li P., Xiong L., Zhao G. (2011). Ginsenoside Rd in experimental stroke: Superior neuroprotective efficacy with a wide therapeutic window. Neurotherapeutics.

[61] Yun Y.J., Park B.H., Hou J., Oh J.P., Han J.H., Kim S.C. (2022). Ginsenoside F1 protects the brain against amyloid beta-induced toxicity by regulating IDE and NEP. Life.

[62] Wang R., Li Y.N., Wang G.J., Hao H.P., Wu X.L., Zhou F. (2009). Neuroprotective effects and brain transport of ginsenoside Rg1. Chin. J. Nat. Med..

[63] Qi L.W., Wang C.Z., Yuan C.S. (2011). Isolation and analysis of ginseng: Advances and challenges. Nat. Prod. Rep..

[64] Thaí T.L., Kang J.M., Lê H.G., Lee J., Yoo W.G., Shin H.J., Sohn W.-M., Na B.-K. (2020). Fowlerstefin, a cysteine protease inhibitor of *Naegleria fowleri*, induces inflammatory responses in BV-2 microglial cells in vitro. Parasit. Vectors.

